# Improved results of primary total hip replacement: Results from the Norwegian Arthroplasty Register, 1987–2007

**DOI:** 10.3109/17453674.2010.537807

**Published:** 2010-11-26

**Authors:** Bjørg-Tilde S Fevang, Stein A Lie, Leif I Havelin, Lars B Engesæter, Ove Furnes

**Affiliations:** ^1^Department of Rheumatology; ^2^The Norwegian Arthroplasty Register, Department of Orthopedic Surgery, Haukeland University Hospital; ^3^Section for Orthopaedic Surgery, Department of Surgical Sciences, University of Bergen; ^4^Department of Health, University Research Bergen, Bergen, Norway

## Abstract

**Background and purpose:**

Over the past 20 years, several changes in treatment policy and treatment options have taken place regarding hip replacement. For this reason, we wanted to investigate the results after hip replacement in terms of revision rate, during a 21-year period among hip replacements reported to the Norwegian Arthroplasty Register.

**Methods:**

110,882 primary total hip replacements were reported to the Norwegian Arthroplasty Register from 1987 through 2007. Risk of revision during the time periods 1993–1997, 1998–2002, and 2003–2007 was compared to that of the reference period 1987–1992. Adjusted Cox regression analyses were performed to compare the risk of revision in different time periods and extended analyses were done to investigate revision within the first postoperative year and after the first year.

**Results:**

There was an overall reduced risk of revision in the time periods 1993–1997, 1998–2002, and 2003–2007 compared to the reference period: RR = 0.81 (95% CI 0.77–0.86), 0.51 (CI 0.47–0.55), and 0.77 (CI 0.68–0.85), respectively. The improved results were due to a marked reduction in aseptic loosening of the femoral and acetabular components in all time periods and in all subgroups of prostheses. A change in the timing of revision took place, with more early revisions and fewer late revisions in the later time periods. Revision due to dislocation and infection increased over time.

**Interpretation:**

The risk of revision decreased during the study period, due to fewer cases of aseptic loosening of prosthetic components. The best results were obtained with the use of cemented prostheses. Prevention of dislocation and infection should be a major goal in the future, as revision due to these causes increased during the study period.

The use of hip arthroplasty surgery has increased with time (Ingvarsson et al. 1999, Wells et al. 2002, Pedersen et al. 2005) as the population ages and patients with more comorbidity and of older age are accepted for surgery. The need for surveillance of long-term results was recognized early, leading to the establishment of national registries. The Swedish Hip Arthroplasty Register was established in 1979, followed by the Finnish equivalent in 1980, and the Norwegian equivalent in 1987.

In one recent publication on trends in primary hip arthroplasty in the USA from 1990 to 2004, an overall decrease in procedure-related complications and adverse diagnoses was seen, although at the same time an increase in the prevalence of comorbidities took place (Liu et al. 2008). Herberts and Malchau found a decrease in revision with time in the Swedish population after hip arthroplasty (Herberts and Malchau 1997).

We evaluated the results of hip arthroplasty, with particular focus on time trends, during the period 1987 through 2007. Overall revision and also revision due to specific causes such as loosening of components, dislocation, or infection were studied. We also studied the timing of an event in relation to the primary operation for given causes of revision, and compared 4 time periods.

## Patients and methods

We used data from the Norwegian Arthroplasty Register (NAR), which is a population-based prospective database (Havelin et al. 2000). All hospitals in Norway at which the procedure is performed (n = 64 in 2007) report to the NAR. Data concerning patient identification, diagnosis, date of surgery, whether the operation was primary or a revision, type of prosthesis, whether cement was used and type of cement, and the use of antibiotics, is derived from a form filled in by the operating surgeon (Furnes et al. 2002). Furthermore, the causes of revision and the procedures performed at revision are reported. In a study from 2006 comparing data from the NAR to the official patient administration system, the data in the NAR were found to be complete (Espehaug et al. 2006).

In the present article these data were used to study changes in the rate of revision arthroplasty after primary surgery, over a 21-year period from 1987 through 2007. The patients were divided into four groups according to the year of primary surgery: 1987–1992, 1993–1997, 1998–2002, and 2003–2007. The first period, from 1987 through 1992, was used as the reference period.

From 1987 through 2007, 110,882 primary total hip replacements were reported to the NAR (Table 1). For the analyses, patients were defined as having osteoarthritis (OA) or not (patients with all other diagnoses), and the analyses were adjusted for diagnosis, sex, and age.

**Table 1.** Age, sex, and diagnosis at primary surgery in 4 time periods

	All	1987–1992	1993–1997	1998–2002	2003–2007
	n (%)	n (%)	n (%)	n (%)	n (%)
Mean age	69	69	70	70	70
Sex (% women)	70	70	70	70	69
n	110,882	24,651	24,659	28,817	32,755
Diagnosis					
Osteoarthritis	79,700 (72)	16,597 (67)	17,130 (70)	20,976 (73)	24,997 (76)
Rheumatoid arthritis	3,465 (3.1)	942 (3.8)	902 (3.7)	851 (3.0)	770 (2.4)
Sequelae after fracture	11,933 (11)	3,299 (13)	3,133 (13)	2,886 (10)	2,615 (8)
Sequelae dysplasia	8,125 (7.3)	2,037 (8.3)	1,795 (7.3)	2,032 (7.1)	2,261 (6.9)
Sequelae dysplasia, total luxation	879 (0.8)	414 (1.7)	214 (0.9)	141 (0.5)	110 (0.3)
Sequelae Perthes'/epiphysiolysis	1,442 (1.3)	319 (1.3)	329 (1.3)	388 (1.3)	406 (1.2)
Ankylosing spondylitis	477 (0.4)	105 (0.4)	116 (0.5)	144 (0.5)	112 (0.3)
Acute fracture	969 (0.9)	55 (0.2)	116 (0.5)	233 (0.8)	565 (1.7)

During the study period, 99 different femur implants, 85 different acetabular implants, and 70 different caput implants were used in Norway (Table 2). The 45 most common implant types used during the study period were published in the annual report from the NAR (http://www.haukeland.no/nrl/eng/, 2008). The Charnley prosthesis (DePuy, Leeds, UK) was the most common implant during the study period and the original monoblock Charnley prosthesis was used throughout the study period.

**Table 2.** The most commonly used implants, in 4 time periods

Most commonly used	1987–1992	1993–1997	1998–2002	2003 and later	Total
a. Acetabular implants					
Charnley (DePuy)	11,947	11,789	10,431	7,270	41,437
Exeter (Stryker)	2,609	2,029	2,866	3,871	11,375
Reflection (Smith & Nephew)	0	818	3,505	6,613	10,936
Titan (DePuy)	1,903	2,262	1,861	1,884	7,910
Elite (DePuy)	597	409	1,261	2,950	5,217
Tropic (DePuy)	1,065	1,443	1,232	82	3,822
Spectron (Smith & Nephew)	2,128	1,117	406	0	3,651
Trilogy (Zimmer)	0	381	1,012	1,517	2,910
SP (Link)	474	393	776	1,211	2,854
b. Femoral implants					
Charnley (DePuy)	12,556	12,291	10,039	6,323	41,209
Exeter (Stryker)	2,652	2,090	3,163	6,304	14,209
Titan (DePuy)	2,669	2,776	3,069	2,913	11,427
Corail (DePuy)	1,096	2,232	2,107	3,392	8,827
Spectron (Smith & Nephew)	51	183	2,552	5,971	8,757
ITH (Smith & Nephew)	1,230	1,446	983	64	3,723
SP (Link)	1	394	582	1,128	2,105
c. Caput implants					
Exeter (Stryker)	2,269	2,088	3,206	6,598	14,161
Universal (Smith & Nephew)	1,652	2,475	3,705	5,898	13,730
Landos (Ortho Medic)	3,824	5,037	2,527	2,566	13,954
Fjord (Ortho Medic)	0	392	2,841	3,431	6,664
SP II Lubinus (Link)	0	388	584	1130	2,102

In prostheses with a cemented femoral component, the caput was modular in 52,371 cases while in 46,447 cases a monoblock femoral component was used. Of the uncemented femoral components, 22,412 modular caput components were used as opposed to only 67 monoblocks.

A prosthesis in which a cemented femoral component and an uncemented acetabular component was used was defined as a hybrid prosthesis, while inverse hybrid was the term used for a prosthesis with an uncemented femoral component and a cemented acetabular component. 23 different cement types were used. These included several variants of the Palacos (Heraeus Medical, Germany; Schering-Plough; Biomet) and Simplex (Howmedica, UK; Stryker) cements, which, for the purposes of this article, are analyzed together.

A revision was defined as the removal or exchange of a part of or the whole implant. On the forms, several causes of revision could be given for the same patient. However, in the present study only one cause was selected for each patient. This was done by a fixed system of priority; for instance, deep infection was selected when it was one of many causes, and pain was only selected when it was the only given cause of revision. Overall revision was defined as revision for any cause.

### Statistics

Information on death or emigration was obtained from Statistics Norway. The patients were followed until time of revision, death, or emigration, or until the end of the study (December 31, 2007), at which point the patients were censored.

Analyses of overall revision (revision for any cause) were done for all prostheses together and separately for cemented, hybrid, inverse hybrid, and uncemented prostheses. A rather homogenous group of patients (n = 28,225) with a Charnley prosthesis cemented with Simplex or Palacos cements was selected in order to investigate possible changes that could not be attributed to changes in implant or cement type. Separate analyses were performed for this group, as well as for a group of all other cemented implants. Charnley prostheses and Palacos and Simplex cements were chosen since they are well documented and have shown good results (Kavanagh et al. 1989, Schulte et al. 1993, Havelin et al. 1995, Furnes et al. 1997, Espehaug et al. 2002, 2009).

Kaplan-Meier survival plots were used to compare cumulative prosthesis survival between subgroups of patients (i.e. patients operated during the 4 time periods). The overall risk of revision and the risk of revision due to specific causes (deep infection, dislocation, or aseptic loosening of the femoral or acetabular component) were calculated using Cox regression analyses. The risk estimates were adjusted for age, sex, and diagnosis. Based on figures for scaled Schoenfeld residuals showing an increased relative risk during the first postoperative year, Cox analyses with time-dependent covariates, with indicators before/after the first postoperative year, were performed. Thus, risk of revision within the first year was compared between the different time periods using Cox regression analysis with time-dependent covariates, as was the risk of revision after the first year (Table 5). All analyses were performed using SPSS software version 17.0.

## Results

**Figure 1.**
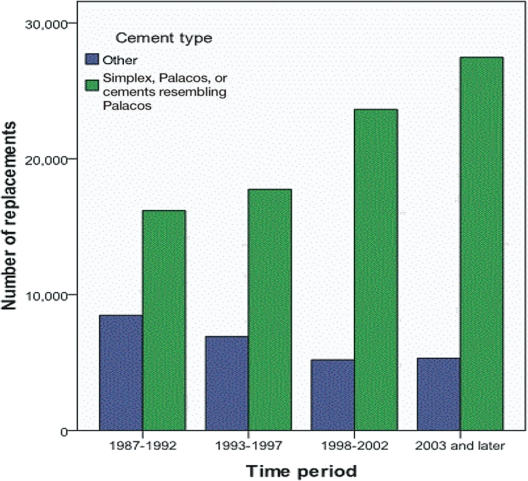
Number of hips inserted using Simplex, Palacos, or cement types resembling Palacos (green), in 4 time periods. Blue represents other cement types.

During the 21-year period, the sex distribution and mean age of the patients remained largely unchanged (Table 1). Subanalyses of age within the group of patients with osteoarthritis showed that the mean age remained the same during the 4 time periods, but the age distribution broadened, with more younger and more older patients operated during the later periods. The cause of arthroplasty changed towards more patients with osteoarthritis and fewer with rheumatoid arthritis and sequelae after fracture (Table 1). Furthermore, there was a change in the types of implant used during the study period, with a decrease in Charnley prostheses in parallel with an increase in the use of Exeter and Spectron femoral components and Reflection cups being the most prominent changes (Table 2a and b). The use of modular caput implants increased in general, and for all the common implant brands (Table 2c). No marked change in the use of cemented versus uncemented prostheses occurred, although there was an increase in the use of inverse hybrid prostheses (with cementation of the acetabular component) (Table 3). The use of the high-viscosity cements Simplex and Palacos—and also cements closely resembling Palacos (Refobacin bone cement R)—increased throughout the study period (Figure 1).

**Table 3.** Fixation method for 4 time periods

	1987–1992	1993–1997	1998–2002	2003 and later	Total
	n (%)	n (%)	n (%)	n (%)	n (%)
Cemented	19,837 (82)	19,833 (81)	23,161 (81)	24,104 (75)	86,935 (79)
Uncemented	3,142 (13)	3,355 (14)	3,715 (13)	4,410 (14)	14,622 (13)
Hybrid ^**b**^	1,028 (4.3)	1,317 (5.4)	1,363 (4.7)	684 (2.1)	4,392 (4.0)
Inverse hybrid ^**c**^	60 (0.2)	45 (0.2)	482 (1.7)	3,140 (9.7)	3,727 (3.4)
Total	24,067 (100)	24,550 (100)	28,721 (100)	32,338 (100)	109,676 (100) **^a^**

**^a^** The fixation method was not reported for 1,206 prostheses.

**^b^** Hybrid means cemented femur component and uncemented cup.

**^c^** Inverse hybrid means uncemented femur component and cemented cup.

**Figure 2.**
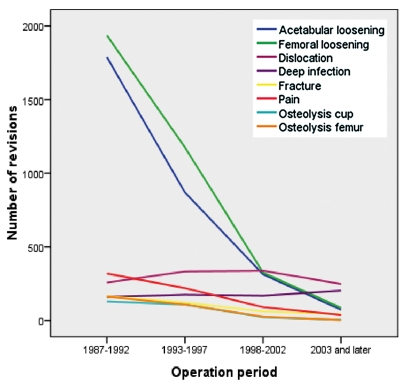
Numbers of revisions for different causes, in 4 time periods.

The major cause of revision was aseptic loosening of one or both implant components, seen in 5,328 cases and constituting 66% of all revision operations (Table 4). Other major causes of revision were dislocation, infection, and pain, constituting 14.5, 8.7, and 8.3 per cent (2.2 with pain as the only cause) of the revisions, respectively (Table 4). A decline in the number of revisions took place throughout the study period, for all causes of revision except dislocation and infection (Figure 2).

**Table 4.** Numbers of revisions due to specific causes for the 4 time periods

Cause of revision	All	1987–1992	1993–1997	1998–2002	2003–2007
Loose femur **^a^**	3,522	1,936	1,175	325	86
Loose acetabulum **^a^**	3,043	1,789	868	313	73
Aseptic loosening **^b^**	5,328	2,984	1,681	526	137
Dislocation	1,177	258	333	338	248
Infection	706	160	175	168	203
Fracture	392	164	123	62	43
Pain	668 (175 **^e^**)	319	220	91	38
Osteolysis acetabulum **^c^**	266	130	109	23	4
Osteolysis femur **^c^**	301	163	109	26	3
Total ^**d**^	8,094	3,806	2,504	1,125	659

**^a^** Aseptic loosening of one component.

**^b^** Aseptic loosening of one or both components.

**^c^** Osteolysis without loosening.

**^d^** More than one cause of revision may be reported; thus, the sum of the different causes does not equal the total number of revision procedures.

**^e^** In 175 patients, pain was the only reported cause of revision.

**Figure 3.**
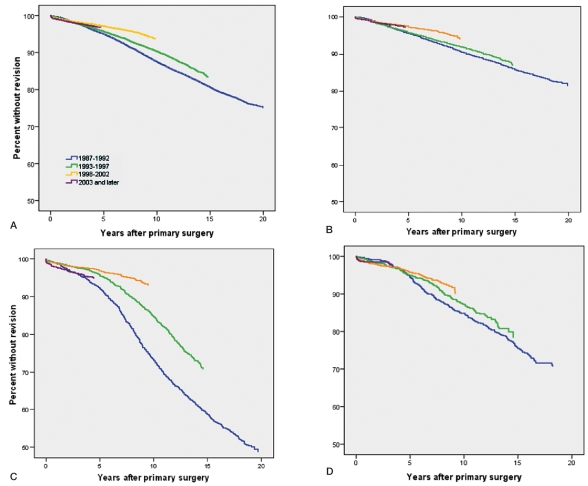
Kaplan-Meier survival plots. Revision for any cause in 4 time periods. A. All prostheses. B. All cemented prostheses. C. All uncemented prostheses. D. All hybrid prostheses.

When considering overall revision (i.e. for any cause) for all types of hip implants together, the risk of revision for patients operated during the second, third, and fourth time periods was lower than for those operated during the reference period (Figure 3a) (RR = 0.51 (95% CI: 0.47–0.55) for 1998–2002 compared to 1987–1992). Early revision, i.e. revision within the first postoperative year, was more frequent in the 3 later time periods (p < 0.001), while the opposite was seen for revisions that took place after the first year (p < 0.001) (Table 5a, first column). This indicates that although the revision rate was falling, revision surgery took place more rapidly after the primary operation in the later periods.

**Table 5a.** Relative risk of overall revision (due to any cause), aseptic loosening of acetabular component, aseptic loosening of femoral component, dislocation, and infection adjusted for age sex, and diagnosis (OA vs. other). All prostheses, n = 101,550

A	B		C		D		E		F	
Total										
1987–1992	1		1		1		1		1	
1993–1997	0.81	<0.001	0.69	<0.001	0.67	<0.001	1.58	<0.001	1.18	0.2
	(0.77–0.86)		(0.63–0.75)		(0.62–0.72)		(1.32–1.90)		(0.94–1.47)	
1998–2002	0.51	<0.001	0.43	<0.001	0.23	<0.001	1.87	<0.001	1.11	0.4
	(0.47–0.55)		(0.37–0.49)		(0.20–0.26)		(1.56–2.26)		(0.88–1.40)	
2003–2007	0.77	<0.001	0.36	<0.001	0.19	<0.001	2.21	<0.001	2.13	<0.001
	(0.68–0.85)		(0.28–0.47)		(0.15–0.24)		(1.80–2.72)		(1.69–2.68)	
First year										
1987–1992	1		1		1		1		1	
1993–1997	1.48	<0.001	1.02	1.0	0.83	0.4	2.22	<0.001	1.99	0.01
	(1.19–1.84)		(0.59–1.75)		(0.54–1.27)		(1.56–3.16)		(1.17–3.41)	
1998–2002	1.64	<0.001	0.89	0.7	0.37	<0.001	2.81	<0.001	2.05	0.007
	(1.33–2.03)		(0.52–1.54)		(0.22–0.63)		(2.01–3.93)		(1.21–3.46)	
2003–2007	2.09	<0.001	0.51	0.04	0.37	<0.001	2.88	<0.001	4.60	<0.001
	(1.71–2.56)		(0.27–0.98)		(0.22–0.63)		(2.06–4.03)		(2.85–7.41)	
After 1 year										
1987–1992	1		1		1		1		1	
1993–1997	0.78	<0.001	0.68	<0.001	0.66	<0.001	1.39	0.002	1.04	0.8
	(0.74–0.83)		(0.63–0.75)		(0.61–0.72)		(1.13–1.72)		(0.80–1.33)	
1998–2002	0.42	<0.001	0.41	<0.001	0.22	<0.001	1.51	<0.001	0.93	0.6
	(0.39–0.46)		(0.36–0.47)		(0.19–0.25)		(1.20–1.91)		(0.71–1.22)	
2003–2007	0.54	<0.001	0.36	<0.001	0.17	<0.001	2.09	<0.001	1.44	0.02
	(0.47–0.62)		(0.27–0.48)		(0.13–0.22)		(1.55–2.83)		(1.06–1.94)	

A Time period B All revisions RR (95% CI) and p–value C Acetabular loosening RR (95% CI) and p–value D Femoral loosening RR (95% CI) and p–value E Dislocation RR (95% CI) and p–value F Infection RR (95% CI) and p–value

Still considering all types of hip implants, the reduction in revisions was due to a statistically significant fall in revisions caused by acetabular or femoral component loosening (Table 5a, second and third columns). For these 2 major causes of revision, no increase in revision within the first postoperative year was seen. The trend was opposite when considering revision due to dislocation, with an overall increase in revisions due to dislocation throughout the study period, with a greater increase in procedures taking place within the first postoperative year, but also after the first year (Table 5a, column 4). A similar trend was seen for revisions due to infection, although it was less evident—except for the period 2003–2007 (Table 5a, column 5).

When analyzing all cemented prostheses separately, the results were very much the same as for all prostheses analyzed together, with an improved overall revision rate (Figure 3b) and particularly less revisions due to aseptic loosening (Table 5b, columns 1, 2, and 3). As was seen in the total group, there were more early revisions and less late revisions during the last 3 time periods. The risk of revision due to dislocation increased during the 3 later time periods, particularly revision procedures performed within the first postoperative year (Table 5b, column 4). Furthermore, revisions due to deep infection increased, as was seen for the total group (Table 5b, column 5).

**Table 5b.** Relative risk of overall revision (due to any cause), aseptic loosening of acetabular component, aseptic loosening of femoral component, dislocation, and infection adjusted for age sex, and diagnosis (OA vs. other). All cemented prostheses, n = 86,929

A	B		C		D		E		F	
Total										
1987–1992	1		1		1		1		1	
1993–1997	0.92	0.01	0.87	0.012	0.81	<0.001	1.57	<0.001	1.17	0.2
	(0.86–0.98)		(0.78–0.97)		(0.75–0.88)		(1.28–1.92)		(0.91–1.49)	
1998–2002	0.59	<0.001	0.64	<0.001	0.25	<0.001	2.01	<0.001	1.07	0.6
	(0.54–0.64)		(0.55–0.74)		(0.22–0.29)		(1.63–2.47)		(0.83–1.37)	
2003–2007	0.72	<0.001	0.40	<0.001	0.17	<0.001	2.20	<0.001	1.82	<0.001
	(0.65–0.81)		(0.30–0.54)		(0.13–0.22)		(1.74–2.77)		(1.41–2.34)	
First year										
1987–1992	1		1		1		1		1	
1993–1997	1.72	<0.001	1.00	1.0	1.08	0.8	2.64	<0.001	2.03	0.01
	(1.34–2.21)		(0.56–1.79)		(0.67–1.74)		(1.74–3.99)		(1.17–3.52)	
1998–2002	1.84	<0.001	0.90	0.7	0.45	0.007	3.51	<0.001	1.84	0.03
	(1.44–2.34)		(0.51–1.60)		(0.25–0.81)		(2.37–5.21)		(1.07–3.18)	
2003–2007	2.01	<0.001	0.36	0.009	0.20	<0.001	3.26	<0.001	3.85	<0.001
	(1.58–2.54)		(0.17–0.77)		(0.09–0.45)		(2.19–4.85)		(2.34–6.34)	
After 1 year										
1987–1992	1		1		1		1		1	
1993–1997	0.87	<0.001	0.86	0.01	0.80	<0.001	1.29	0.04	1.01	1.0
	(0.81–0.94)		(0.77–0.97)		(0.74–0.87)		(1.02–1.64)		(0.76–1.32)	
1998–2002	0.49	<0.001	0.62	<0.001	0.25	<0.001	1.53	0.001	0.91	0.5
	(0.45–0.54)		(0.54–0.73)		(0.21–0.28)		(1.18–1.99)		(0.68–1.21)	
2003–2007	0.52	<0.001	0.42	<0.001	0.17	<0.001	2.02	<0.001	1.23	0.2
	(0.44–0.60)		(0.31–0.58)		(0.13–0.23)		(1.44–2.81)		(0.88–1.71)	

A–F, see Table 5a.

**Figure 4.**
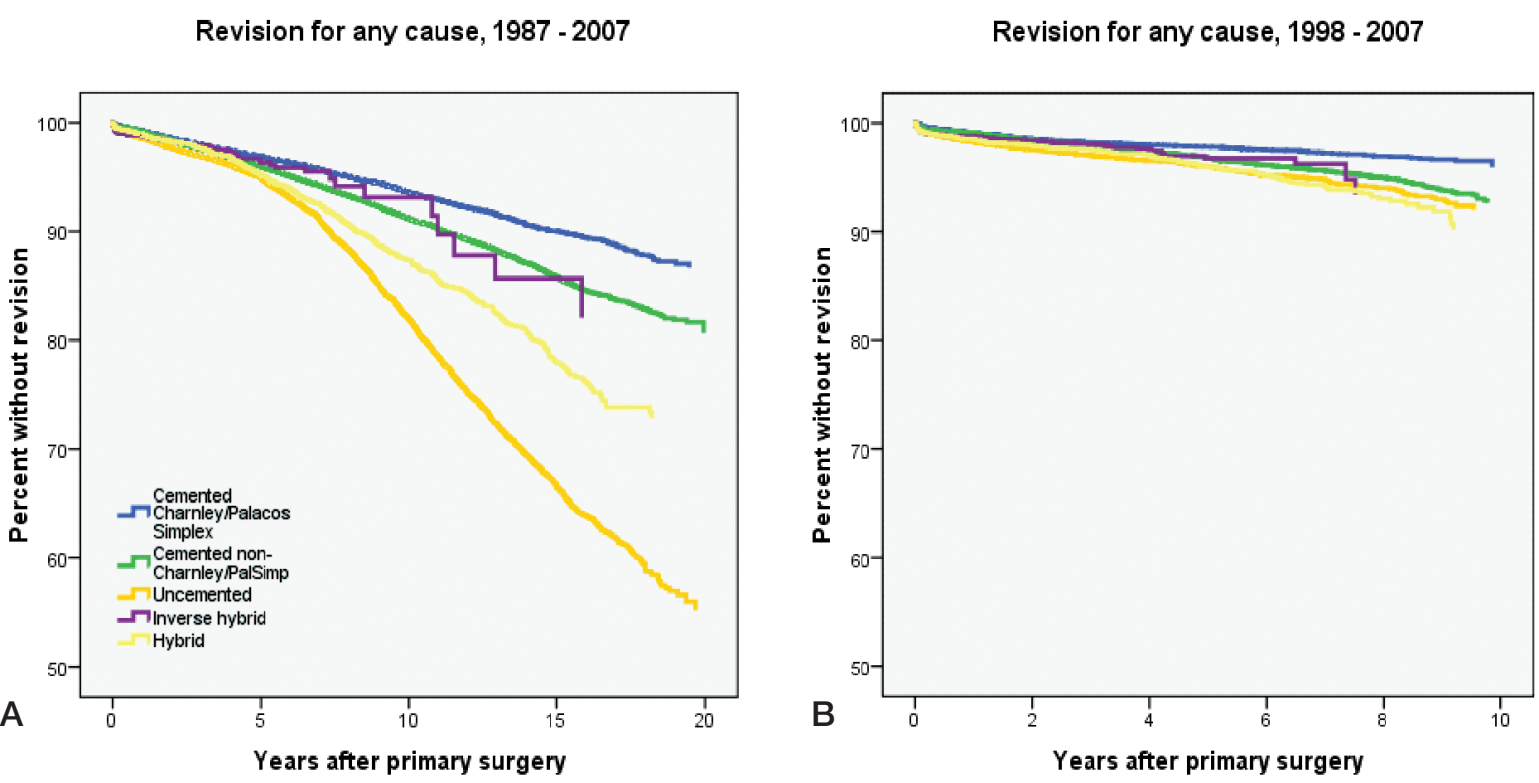
Kaplan-Meier survival plot. Revision for any cause. A. 1987–2007. B. 1998–2007.

Patients with cemented Charnley prostheses (with Palacos or Simplex cement) had a better overall survival compared to those with uncemented, hybrid, inverse hybrid, and non-Charnley cemented prostheses (p < 0.001) (Figure 4a), a finding that was less prominent but still present during the last 10-year period (p<0.001) (Figure 4b). In the 1,206 patients for whom the fixation method was unknown, the results closely resembled those of the hybride group (not included in Figure 4). In the adjusted analysis, overall revision rate for the Charnley group increased during the second period compared to the first, while a statistically significant decrease in revision was seen in the third period, with a similar trend during the fourth one (Table 5c, column 1). Revisions performed within the first year were more common during all the 3 later periods, while the opposite was seen for revisions after the first year (except in the second time period).

**Table 5c.** Relative risk of overall revision (due to any cause), aseptic loosening of acetabular component, aseptic loosening of femoral component, dislocation, and infection adjusted for age sex, and diagnosis (OA vs. other). Charnley prostheses with Palacos or Simplex cement, n = 28,225

A	B		C		D		E		F	
Total										
1987–1992	1		1		1		1		1	
1993–1997	1.27	<0.001	1.12	0.3	1.28	0.001	1.75	0.002	1.15	0.5
	(1.13–1.44)		(0.89–1.40)		(1.11–1.49)		(1.23–2.50)		(0.78–1.70)	
1998–2002	0.61	<0.001	0.44	<0.001	0.21	<0.001	2.21	<0.001	0.88	0.6
	(0.51–0.72)		(0.30–0.63)		(0.15–0.28)		(1.54–3.18)		(0.58–1.35)	
2003–2007	0.88	0.3	0.31	0.003	0.25	<0.001	2.61	<0.001	1.76	0.02
	(0.70–1.12)		(0.14–0.67)		(0.14–0.45)		(1.68–4.07)		(1.11–2.79)	
First year										
1987–1992	1		1		1		1		1	
1993–1997	2.01	0.002	0.97	1.0	1.10	0.8	2.78	0.003	1.49	0.3
	(1.30–3.09)		(0.33–2.89)		(0.46–2.61)		(1.42–5.45)		(0.66–3.34)	
1998–2002	2.00	0.002	0.91	0.9	0.61	0.3	3.30	<0.001	1.50	
	(1.23–3.07)		(0.31–2.72)		(0.23–1.63)		(1.71–6.39)		(0.68–3.35)	
2003–2007	2.31	<0.001	0.24	0.2	0.17	0.09	3.63	<0.001	2.89	0.01
	(1.45–3.68)		(0.03–2.02)		(0.021.30)		(1.79–7.40)		(1.30–6.43)	
After 1 year										
1987–1992	1		1		1		1		1	
1993–1997	1.22	0.002	1.13	0.3	1.29	0.001	1.42	0.1	1.06	0. 8
	(1.07–1.39)		(0.89–1.42)		(1.11–1.50)		(0.93–2.17)		(0.68–1.66)	
1998–2002	0.46	<0.001	0.39	<0.001	0.18	<0.001	1.83	0.008	0.70	0.2
	(0.38–0.56)		(0.26–0.58)		(0.13–0.26)		(1.17–2.87)		(0.41–1.17)	
2003–2007	0.62	0.004	0.33	0.009	0.27	<0.001	2.36	0.009	1.34	0.3
	(0.45–0.86)		(0.14–0.76)		(0.15–0.48)		(1.24–4.50)		(0.74–2.45)	

A–F, see Table 5a.

When considering revision due to aseptic loosening, the risk of revision within the first postoperative year remained unchanged throughout the study period, while a reduction in such revisions performed after the first postoperative year was seen after 1997 (Table 5c, columns 2 and 3). As for the total group, the risk of revision due to dislocation increased during the study period, but no significant change in the risk of revision due to deep infection was seen except for an increased risk in the period from 2003 through 2007 (Table 5c, columns 4 and 5).

The results after insertion of cemented prostheses other than Charnley were similar to those described for the total study group (Table 5d).

**Table 5d.** Relative risk of overall revision (due to any cause), aseptic loosening of acetabular component, aseptic loosening of femoral component, dislocation, and infection adjusted for age sex, and diagnosis (OA vs. other). Other cemented prostheses, n = 58,704

A	B		C		D		E		F	
Total										
1987–1992	1		1		1		1		1	
1993–1997	0.83	<0.001	0.86	0.02	0.68	<0.001	1.50	0.002	1.15	0.4
	(0.76–0.90)		(0.75–0.97)		(0.61–0.75)		(1.17–1.94)		(0.84–1.58)	
1998–2002	0.60	<0.001	0.72	<0.001	0.27	<0.001	1.94	<0.001	1.17	0.3
	(0.55–0.66)		(0.61–0.85)		(0.24–0.32)		(1.51–2.51)		(0.86–1.60)	
2003–2007	0.67	<0.001	0.41	<0.001	0.15	<0.001	2.08	<0.001	1.90	<0.001
	(0.59–0.76)		(0.30–0.56)		(0.11–0.20)		(1.58–2.73)		(1.40–2.57)	
First year										
1987–1992	1		1		1		1		1	
1993–1997	1.60	0.003	1.07	0.8	1.11	0.7	2.53	0.001	2.47	0.02
	(1.17–2.19)		(0.54–2.14)		(0.62–1.98)		(1.49–4.29)		(1.16–5.25)	
1998–2002	1.79	<0.001	0.93	0.8	0.39	0.01	3.67	<0.001	2.09	0.05
	(1.34–2.40)		(0.47–1.81)		(0.19–0.81)		(2.25–5.99)		(0.99–4.40)	
2003–2007	1.90	<0.001	0.36	0.02	0.20	<0.001	3.20	<0.001	4.78	<0.001
	(1.43–2.50)		(0.16–0.84)		(0.08–0.49)		(1.97–5.20)		(2.46–9.31)	
After 1 year										
1987–1992	1		1		1		1		1	
1993–1997	0.79	<0.001	0.85	0.02	0.67	<0.001	1.26	0.1	0.96	0.8
	(0.73–0.86)		(0.75–0.97)		(0.60–0.74)		(0.94–1.69)		(0.67–1.36)	
1998–2002	0.52	<0.001	0.71	<0.001	0.27	<0.001	1.41	0.04	1.03	0.9
	(0.47–0.58)		(0.60–0.84)		(0.23–0.31)		(1.03–1.95)		(0.73–1.46)	
2003–2007	0.48	<0.001	0.43	<0.001	0.14	<0.001	1.88	0.002	1.20	0.4
	(0.40–0.56)		(0.30–0.61)		(0.10–0.20)		(1.27–2.78)		(0.81–1.79)	

A–F, see Table 5a.

Uncemented prostheses (n = 14,621) generally had a worse prognosis than the cemented ones (Figure 4a), but the results improved with time and the gap between cemented and uncemented implants narrowed (Figure 4b). The regression analysis showed a reduction in overall revision with RR = 0.3 (95% CI: 0.25–0.35) when comparing the period 1998–2002 to the reference period (Table 5e, first column), also illustrated in Figure 3c. However, the overall revision rate as well as revision due to component loosening increased during the last study period (2003–2007) compared to 1998–2002 (Table 5e, columns 1–3). Furthermore, the risk of dislocation increased in this prosthesis group as well as for the cemented ones, particularly during the last study period (Table 5e, column 4) and the risk of revision due to infection increased markedly in the last study period (RR = 5.5 (95% CI: 2.9–11).

**Table 5e.** Relative risk of overall revision (due to any cause), aseptic loosening of acetabular component, aseptic loosening of femoral component, dislocation, and infection adjusted for age sex, and diagnosis (OA vs. other). Uncemented prostheses, n = 14,621

A	B		C		D		E		F	
Total										
1987–1992	1		1		1		1		1	
1993–1997	0.61	<0.001	0.46	<0.001	0.16	<0.001	1.56	0.02	1.31	0.4
	(0.55–0.67)		(0.40–0.53)		(0.12–0.22)		(1.06–2.28)		(0.69–2.48)	
1998–2002	0.30	<0.001	0.09	<0.001	0.11	<0.001	1.28	0.3	1.51	0.4
	(0.25–0.35)		(0.06–0.14)		(0.07–0.17)		(0.83–1.95)		(0.77–2.96)	0.2
2003–2007	0.91	0.4	0.28	<0.001	0.27	<0.001	1.93	0.005	5.51	<0.001
	(0.75–1.11)		(0.15–0.52)		(0.17–0.45)		(1.22.–3.05)		(2.85–10.66)	
First year										
1987–1992	1		1		1		1		1	
1993–1997	0.87	0.6	1.26	0.8	0.34	0.04	1.25	0.5	1.85	0.6
	(0.55–1.38)		(0.28–5.63)		(0.12–0.93)		(0.63–2.49)		(0.17–20.42)	
1998–2002	1.04	0.9	0.59	0.6	0.19	0.01	1.15	0.7	6.00	0.09
	(0.67–1.61)		(0.10–3.53)		(0.06–0.67)		(0.58–2.28)		(0.74–48.81)	
2003–2007	2.08	<0.001	1.41	0.6	0.75	0.5	1.68	0.1	18.07	0.005
	(1.41–3.05)		(0.34–5.90)		(0.35–1.64)		(0.89–3.16)		(2.43–134.2)	
After 1 year										
1987–1992	1		1		1		1		1	
1993–1997	0.60	<0.001	0.46	<0.001	0.15	<0.001	1.71	0.02	1.27	0.5
	(0.54–0.66)		(0.40–0.53)		(0.11–0.21)		(1.08–2.70)		(0.65–2.46)	
1998–2002	0.25	<0.001	0.09	<0.001	0.11	<0.001	1.33	0.3	1.12	0.8
	(0.20–0.30)		(0.06–0.13)		(0.07–0.17)		(0.78–2.28)		(0.53–2.37)	
2003–2007	0.62	0.001	0.18	<0.001	0.14	<0.001	2.11	0.04	3.77	0.001
	(0.47–0.83)		(0.08–0.42)		(0.07–0.31)		(1.04–2.27)		(1.70–8.38)	

A–F, see Table 5a.

The use of hybrid prostheses decreased in the latest time period while the opposite was seen for inverse hybrids (Table 3). The survival curve for hybrids showed an improvement similar to that of other types, and even for the last time period, the development appeared to follow that of the previous time period with more early revisions and fewer late ones, although a longer follow-up period would be needed to ascertain this (Figure 3d).

**Figure 5.**
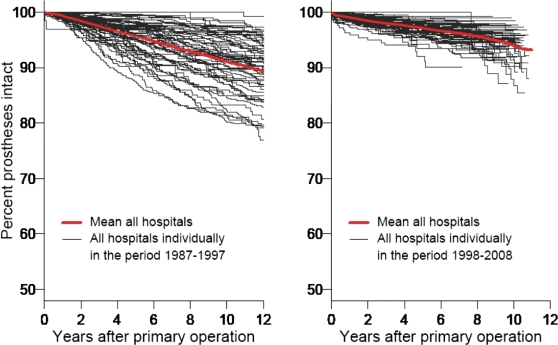
Kaplan-Meier survival plot for cemented hip prostheses: every hospital in Norway in 2 time periods. A. 1987–1997. B. 1998–2008.

Kaplan-Meier survival curves for every hospital performing hip arthroplasties in Norway during the 2 time periods 1987–1997 and 1998–2007 showed a general improvement—leading to a narrower cluster of curves—with the mean survival for the latter time period being higher than for the first one (Figure 5).

## Discussion

Our major finding was a general improvement in the results of hip replacement surgery during the 21-year period. This was seen for the total group as well as for all subgroups studied. Similar findings were reported from the Swedish Hip Registry (Herberts and Malchau 1997). However, in a recently published study on hip and knee arthroplasty in the USA performed 1990–2002, Kurtz et al. (2005) found a constant revision rate during the study period. The positive development in Norway was due to a fall in revisions for aseptic loosening, a finding that has also been reported from the Swedish register (Herberts and Malchau 2000).

An important cause of the improvement in results seen in our study can be attributed to the increasing use of well-documented implants with good results. We believe that the publication of registry studies pointing out inferior implants and cements has played an important role in this development. For example, articles from the NAR concerning the role of the cement type used for fixation were published in 1995, 1997, and in 2002 (Havelin et al. 1995, Furnes et al. 1997, Espehaug et al. 2002). In these studies, the superiority of high-viscosity cements was documented and use of the inferior Boneloc cement ceased as a result, as did the use of CMW cements. The benefit of antibiotic-loaded cement and prophylactic systemic antibiotics was also demonstrated in studies from the registry (Espehaug et al. 1997, Engesaeter et al. 2003). Courses in cementation technique may also have contributed to this improvement. Furthermore, certain brands of uncemented acetabular and femoral components have been shown to be associated with an increased risk of loosening (Havelin et al. 1995, Havelin et al. 1995, 2002, Hallan et al. 2007), which may have contributed to the improvement in the results of uncemented prostheses found in the period 1998–2002.

National registries provide valuable information on the epidemiology of specific diagnoses and/or treatments, treatment results, and time changes in treatment and results. The Norwegian Arthroplasty Register has data concerning hip arthroplasty from 1987 to the present. Data from the register is made public through annual reports, which are available on the internet (www.haukeland.no/nrl). These data thus represent performance measurements for hip arthroplasty in Norway, which is important to surgeons regarding the choice of implant type, fixation method, surgical access, and the use of antibiotics. Furthermore, such information may be of use to health policy makers in providing information on present and future demands (Kurtz et al. 2007), and also to the hospitals since results for each individual hospital (compared to all the others) are sent to all participating hospitals.

The positive development in terms of a declining revision rate was, however, also seen in the rather homogenous subgroup of Charnley implants inserted with Simplex or Palacos cement, although the improvement was not seen until the third time period (1998–2002). This indicates that improvement in surgical technique may also have played a part in the improvement in revision rate, which was indeed the intention when obligatory training programs in prosthesis surgery were initiated for surgeons in 1995. The Norwegian company selling the Charnley prosthesis also initiated training programs for surgeons in 1995, emphasizing modern cementing technique and the same company introduced new instruments for improved prosthesis placement.

In addition to the major finding of improved results during the 21-year study period, several other changes took place. The indication for inserting a hip implant changed, in that a gradual increase in the percentage of patients operated due to osteoarthritis was seen throughout the study period. Furthermore, the patients operated before 1993 were younger, which may, in part, be related to the first point. However, after 1993 the mean age remained stable. The percentage of patients operated due to osteoarthritis increased, but the mean age in this patient group remained the same due to the acceptance of both older patients and younger patients for hip replacement surgery. A change in the timing of revision surgery took place, with more early and fewer late revisions in the latter time periods. This might reflect a change in failure types, with more revisions due to dislocation and early infections and fewer late revisions due to aseptic loosening.

Another important trend was how the difference in results for the different hospitals diminished, giving a generally better hip arthroplasty service due to changes in the choice of implant brand, fixation method, and cement type. For instance, the use of inverse hybrid prostheses (with cementation of the acetabular component) increased, a method that gave better results than uncemented prostheses and the regular hybrids. The changes in implant and cement types and brands may, in part, have resulted from published results from register studies.

A newly published nationwide study from the USA has also found an increase over a 15-year period in the incidence of hip arthroplasties diagnosed with infection (Kurtz et al. 2008). However, a British study of about 6,000 hip arthroplasties reported no change in the rate of prosthetic joint infections (Phillips et al. 2006). We found an increase in revisions due to deep infection that was most pronounced for uncemented prostheses. Hip prosthesis surgery in patients with more comorbidity, and more patients being on immunosuppressive drugs, may be plausible explanations for this development. For instance, obesity and diabetes are known risk factors for infection, and the incidences of both conditions are increasing in the population. In addition, an increased awareness of low-virulence infections may have caused more surgeons to be aware of this problem. Consequently, some revision operations that would previously have been designated “revision due to aseptic loosening” are now correctly characterized as infections. Changes in treatment policy, as described by Dale et al. in a recently published study from our registry, may also have influenced the risk of prosthesis infection (Dale et al. 2009). For example, an increase in patients with higher ASA score was seen during the most recent years of the study (this factor was registered in the NAR from 2005), and higher ASA scores adversely influenced the rate of infection. On the other hand, antibiotic-loaded bone cement protects against infection (Engesaeter et al. 2003, Dale et al. 2009) and the increasing use of antibiotic-loaded cement thus tended to reduce the risk of revision due to infection.

In the large American study, dislocation was the most common cause of revision surgery (Kurtz et al. 2008). In Norway, loosening of prosthesis components was by far the most frequent cause of revision, but the risk of revision caused by dislocation increased. More revisions due to dislocation may result from more young patients having hip implants, as they are more active. Increasing use of small femoral heads on modular prostheses has been shown to be associated with an increased risk of revision due to dislocation (Bystrom et al. 2003, Berry et al. 2005). In the study by Bystrom et al. on data from the NAR, the use of 28-mm heads in the later period, compared to 32-mm heads previously, was found to be the main cause of the increase in revisions due to dislocation. From 2006, the use of 32-mm, 36-mm, and even larger head sizes has increased in both older and younger patients, in combination with the use of new bearing surfaces such as highly crosslinked polyethylene. Hopefully, this will contribute to a reduction in revisions due to dislocation in the future. Furthermore, the surgical approach has been shown to influence the risk of dislocation (Berry et al. 2005, Arthursson et al. 2007). In the article by Arthursson et al. from Norway, the change from a lateral approach with trochanteric osteotomy to a lateral approach without trochanteric osteotomy contributed to the increase in dislocation rate for Charnley prostheses. Possible changes in treatment policy based on these findings might, in time, lead to a decline in the rate of revisions due to dislocation.

In conclusion, from 1987 through 2007, the revision rate after hip arthroplasty surgery decreased. The best results were obtained with the use of cemented prostheses, but the results of uncemented prostheses also improved throughout the study period. A change in the revision pattern took place, with more early revisions and less late revisions performed. There was an increase in revisions due to dislocation and infection.
